# Supplementary material for: Knowledge, attitude, and practice of antenatal exercises among pregnant women in Ethiopia: A cross-sectional study
Source: PLoS One. 2021 Feb 19;16(2):e0247533. doi: 10.1371/journal.pone.0247533 (PMC7895387; doi:10.1371/journal.pone.0247533)
Supplement: S1 File — (DOCX) [file pone.0247533.s001.docx]

# Knowledge, Attitude, and Practice of antenatal exercise of pregnant women, Gondar, UoGCSH, Ethiopia

**SURVEY INFORMATION: Antenatal exercise KAP Questionnaire ID:**

| **Location and Date, Consent, ID** | | | **Response** | | | | **Code** |
| --- | --- | --- | --- | --- | --- | --- | --- |
|  | Interviewer ID | |  | | | | SD1 |
|  | Date of Data collection | |  | | | | SD2 |
|  | Consent has been read and obtained | | 1. Yes, 2. No **If No, End** | | | | SD3 |
|  | Time taken for interview | | In minutes: | | | | SD4 |
| 5. | Are you pregnant/period of amenorrhea for 20 weeks | | 1. Yes 2 , No **If no end** | | | | SD5 |
| 6. | Are you medically advised to minimize physical activity or rest/previous risk pregnancy? | | 1. Yes 2. No | | | | SD6 |
| 7. | What is your age? | |  | | | | SD7 |
| 8. | What is the current month of gestation? | |  | | | | SD8 |
| 9. | How would you describe your socio-economic status [family etb/month] | | 1. High income, 2. Middle income, 3. Low | | | | SD9 |
| 10. | What is the highest level of education you have completed? | | 1. No formal education 2 .Primary school   3.Secondary school 4.Diploma 5. Degree and above | | | | SD10 |
| 11. | What is your religion? | | 1. Orthodox christian 2. Protestants 3. Muslim 4.Other | | | | SD11 |
| 12. | What is your *ethnic group?* | |  | | | | SD12 |
| 13. | Where you come from? | | 1. Urban 2. Rural | | | | SD13 |
| 14. | What is your marital status? | | 1.Currently married 2. Separated 3. Divorced 4. Widowed 5. Cohabitating | | | | SD14 |
| 15. | Are you currently employed | | 1. Yes 2. No *(If no, go to ques # 17 & 18 )* | | | | SD15 |
| 16. | What is your occupation? | |  | | | | SD16 |
| 17. | If yes, for how long in years/months | |  | | | | SD17 |
| 18. | If yes, to which category your work belongs | | 1. Self-employment , 2. Private 2. Governmental | | | | SD18 |
| 19. | How many members do you have in your family? | |  | | | | SD19 |
| 20. | What type of family is yours? | | 1. Extended family 2. Nuclear family | | | | SD20 |
| 21. | Is this your first pregnancy? | | 1. Yes, 2. No | | | | SD21 |
| 22. | If no, How many previous successful pregnancy to had? | |  | | | | SD22 |
| 23. | No of abortions | |  | | | | SD23 |
| 24. | Number of children you have? | |  | | | | SD24 |
| 25. | What was the mode of delivery? | | Labour______, Caesarean________ | | | | SD25 |
| 26. | Where were the place of delivery? | |  | | | | SD26 |
| 27. | Have you been advised to perform walking as an exercise? | | 1. Yes, 2. No | | | | SD27 |
| 28. | Have you been advised to perform ankle-toe movement? | | 1. Yes, 2. No | | | | SD28 |
| 29. | Have you been advised to do ante-natal exercise? | | 1. Yes, 2. No | | | | SD29 |
| 30. | Are you practising exercise during P? | | 1. Yes, 2. No | | | | SD30 |
| 31. | Have you ever been engaged in physical activity before? | | 1. Yes, 2. No | | | | SD31 |
| 32. | Are you suffering/been diagnosed with any of this? | | 1. DM 2. GDM 3. HTN 4.UI 2. LBP 6. Varicose vein 7. Weight gain | | | | SD32 |
| 33. | Weeks of pregnancy | |  | | | | SD33 |
| 34. | Have you ever smoked? | | 1. Yes 2. No | | | | SD34 |
| 35. | If yes, Do you smoke during P? | | 1. Yes 2. No | | | | SD35 |
| 36. | Do you drink alcohol during P? | | 1. Yes 2. No | | | | SD36 |
| 37. | Height | | In cm | | | | SD37 |
| 38. | Weight | | In kg | | | | SD38 |
|  | | | | | | |  |
| 39 | | **KNOWLEDGE** | | **Yes** | **I don’t know** | **No** | **Code** |
| 40 | | Have you ever heard about ante-natal exercise? | |  |  |  | K1 |
| 41 | | Have you ever heard about breathing exercise? | |  |  |  | K2 |
| 42 | | Have you ever heard about back exercise? | |  |  |  | K3 |
| 43 | | Have you ever heard about abdominal exercise? | |  |  |  | K4 |
| 44 | | Have you ever heard about ankle-toe exercise? | |  |  |  | K5 |
| 45 | | Have you ever heard about aerobics? | |  |  |  | K6 |
| 46 | | Have you ever heard about yoga? | |  |  |  | K7 |
| 47 | | Have you ever heard about cycling? | |  |  |  | K8 |
| 48 | | Exercise during P should be individualized | |  |  |  | K9 |
| 49 | | Do you know pelvic floor strengthening exercises? | |  |  |  | K10 |
| 50 | | Do you know abdominal muscles strengthening exercise? | |  |  |  | K11 |
| 51 | | **KNOWLEDGE ---BENEFIT** | |  |  |  | **Code** |
| 52 | | Exercise reduces risk of back pain during pregnancy | |  |  |  | KB1 |
| 53 | | Prevents excessive weight gain in pregnancy | |  |  |  | KB2 |
| 54 | | Strengthens pelvic floor muscle in P | |  |  |  | KB3 |
| 55 | | Reduces the risk of GDM | |  |  |  | KB4 |
| 56 | | Increases energy and stamina during P | |  |  |  | KB5 |
| 57 | | Better ability to cope with labour and delivery | |  |  |  | KB6 |
| 58 | | More rapid post-natal recovery | |  |  |  | KB7 |
| 59 | | **KNOWLEDGE-PRECAUTION** | |  |  |  | **Code** |
| 60 | | Drink plenty of water before exercise | |  |  |  | KP1 |
| 61 | | Drink plenty of water during exercise | |  |  |  | KP2 |
| 62 | | Do exercise outside when it is humid | |  |  |  | KP3 |
| 63 | | Standing or lie on back during exercise | |  |  |  | KP4 |
| 64 | | Drink a plenty of caffeine | |  |  |  | KP5 |
| 65 | | Drink much of alcohols | |  |  |  | KP6 |
| 66 | | **KNOWLEDGE ----CONTRA-INDICATIONS** | |  |  |  | Code |
| 67 | | Chest pain during P | |  |  |  | KC1 |
| 68 | | Difficulty in breathing during P | |  |  |  | KC2 |
| 69 | | Abdominal pain during P | |  |  |  | KC3 |
| 70 | | Back pain during P | |  |  |  | KC4 |
| 71 | | Diabetes during P | |  |  |  | KC5 |
| 72 | | HTN during P | |  |  |  | KC6 |
| 73 | | Uterine contractions during P | |  |  |  | KC7 |
| 74 | | Vaginal bleeding during P | |  |  |  | KC8 |
| 75 | | Premature labour during P | |  |  |  | KC9 |
| 76 | | Headache during P | |  |  |  | KC10 |
| 77 | | Dizziness during P | |  |  |  | KC11 |
| 78 | | Decreased fetal movement during P | |  |  |  | KC12 |
| 79 | | Anemia during P | |  |  |  | KC13 |
|  | | | | | |  | |

|  | **Attitudes regarding antenatal exercise** | **Yes** | **No** | **Code** |
| --- | --- | --- | --- | --- |
| 80 | Do you think, doing Exercise during pregnancy is essential |  |  | A 1 |
| 81 | Doing Exercise during pregnancy reduce prevent from complications |  |  | A 2 |
| 82 | Do you think regular exercise facilitates normal delivery |  |  | A 3 |
| 83 | Do you think exercise will help you recovery soon after delivery |  |  | A 4 |
| 84 | Do you feel that prescribed exercises are safe for your baby |  |  | A5 |
| 85 | Do you think that exercising during pregnancydoes not suit our culture |  |  | A6 |
| 86 | Any pregnant mother can perform exercises without the advices and recommendations of healthcare professionals |  |  | A7 |
| 87 | During pregnancy the priority should be improvement of nutrition and the rest and not physical exercises |  |  | A8 |
| 88 | Performing day to day household activities gives adequate physical exercises to pregnant women and they do not have to perform recommended exercises during pregnancy |  |  | A 9 |
| 89 | Do you think you will feel energetic, doing exercise? |  |  | A 10 |
| 90 | Do you personally like doing exercise? |  |  | A 11 |
| 91 | Do you have enough time to do exercise daily? |  |  | A 12 |
| 92 | Do you think ANEx are safe to practice? |  |  | A 13 |
| 93 | Have you ever been advised to do exercise |  |  | A 14 |
| 94 | Do you have sufficient information on ex during P |  |  | A 15 |
| 95 | Do you get enough family support for doing exercise? |  |  | A 16 |

|  | **PRACTICE** | | **Score 2/ excellent:** Performs exs for > 3times/week | **Score 1/Good**: Performs exs for < 3 times/week | | **Score 0/poor**: Not at all | **Code** |
| --- | --- | --- | --- | --- | --- | --- | --- |
| 96 | Walking | |  |  | |  | P 1 |
| 97 | Ankle, toe exercises | |  |  | |  | P 2 |
| 98 | Abdominal strengthening exercises | |  |  | |  | P 3 |
| 99 | Pelvic floor strengthening exercises | |  |  | |  | P 4 |
| 100 | Breathing exercises | |  |  | |  | P 5 |
| 101 | Relaxation exercises | |  |  | |  | P 6 |
| **Barriers to practice ANEx** | | | | | | |  |
| 102 | What were the barriers that prevents you from practicing ANEx? *Tick boxes*  (multiple response possible) | Lack of time  Lack of information/training  Harmful to foetus  Lack of family support | | | Feel tried  Don’t like exercising  Family advice not to  Uncomfortable during pregnancy | | **PB1** |

**Qualitative:** a. what is your perspective of being physically active or adapting exercise habits during pregnancy? (Write the exact words reported)

**_____________________________________________________________**

**Thank you for time and participation!**
