# Supplementary material for: Knowledge, attitude, and practice of antenatal exercises among pregnant women in Ethiopia: A cross-sectional study
Source: PLoS One. 2021 Feb 19;16(2):e0247533. doi: 10.1371/journal.pone.0247533 (PMC7895387; doi:10.1371/journal.pone.0247533)
Supplement: S2 File — (DOCX) [file pone.0247533.s002.docx]

# Title: Antenatal exercises during pregnancy: knowledge, attitude, and practice of pregnant women in Ethiopia: a cross-sectional study

# STROBE Statement—checklist of items that should be included in reports of *cross-sectional studies*

|  | | Item no | | Recommendation |
| --- | --- | --- | --- | --- |
| **Title and abstract** | | 1 | | 1. Study design is indicated in the title, abstract, methods section as cross-sectional study design. |
|  |  |  |  | (b) A balanced summary of what was done and what was found is provided in the methods and result section of the abstract section. |
| Introduction | | | | |
| Background/rationale | | 2 | | The scientific background and rationale for conducting this study is reported under background section in this manuscript. |
| Objectives | | 3 | | The general and specific objectives of this study stated in the last paragraph under background section. |
| Methods | | | | |
| Study design | | 4 | | This study used institutional-based cross-sectional design and the same is mentioned 1^st^ paragraph of method section. |
| Setting | | 5 | | A detailed description of the study area, sample catchment area, location, population attendance in the institution, and participant, described in the 1^st^ and 2^nd^ paragraph of method section under *study design, setting, and population* sub-section. |
| Participants | | 6 | | Pregnant mothers (aged 18 and 49 years) attending antenatal care at the ANC clinic, UoGCSH, Gondar, Ethiopia. *3^rd^ Paragraph Method section.* |
| Variables | | 7 | | Outcome variables (knowledge, attitude, and practice of antenatal exercise) and predictor variables are operationally defined under *methods section, data collection tools, in the 4^th^ paragraph.* |
| Data sources/ measurement | | 8 | | Source of data and *data analysis methods are discussed in the 5^th^ paragraphs of methods section*, under sub-section data processing and data analysis sub-section. |
| Bias | | 9 | | Efforts to address potential sources of bias were described in several parts of methods section and discussion section under limitations. |
| Study size | | 10 | | Sample size determination, assumptions, and sampling technique are mentioned under *sample size determination and sampling technique sub-section under methods*. |
| Quantitative variables | | 11 | | All quantitative variables (scores of KAP) treated as qualitative after categorizing (based on cut off) them in one of most commonly used categories and category used are mentioned under *data collection tool and methods*. |
| Statistical methods | | 12 | | (*a*) Statistical methods used in this study are described under data analysis sub-section in the last paragraph of method session. |
|  |  |  |  | (*b*) Both sub group analysis and interaction terms were used. |
|  |  |  |  | (*c*) There were no missing data in this study |
|  |  |  |  | *(d)* Not applicable |
|  |  |  |  | (*e*) Not applicable |
| Results | | | | |
| Participants | 13 | | (a) Number of pregnant mothers, response rate, and characteristics are presented in the 1^st^ and 2^nd^ paragraph of results section and the detail socio-demographic characteristics in the table 1 and text under results. | |
|  |  |  | (b) About 82.5% pregnant women participated (90% of power calculated sample) and the most common reason for non-response was not being interested in interview. | |
|  |  |  | (c) This was cross-sectional study so; there is no flow as that of longitudinal study. | |
| Descriptive data | 14 | | (a) Characteristics of study participants (e.g. demographic, maternal, awareness, knowledge, attitude, and practice) and information on potential confounders is presented in tables 1. | |
|  |  |  | (b) There were no missing data in this study | |
| Outcome data | 15 | | Outcome variable KAP (scoring methods, cut-off used, categorising) described and summarized in table 2-4. | |
| Main results | 16 | | (*a*) Unadjusted estimates and confounder-adjusted estimates and their precision (eg, 95% confidence interval) are presented in table 5. Discussed in 1^st^paragraph under regression analysis of result section. | |
|  |  |  | 1. Category boundaries of continuous variables were categorized and reported in all tables. | |
|  |  |  | (*c*) Regression model was used and expressed in odds ratio. | |
| Other analyses | 17 | | When a clear sub group existed, analyses were performed and interaction terms were used to examine potential association . | |
| Discussion | | | | |
| Key results | 18 | | Key results to study objectives are discussed under discussion session with references. | |
| Limitations | 19 | | Limitations and possible strengths related to this study are discussed in the final paragraph of discussion session on the way of viewing direction for researchers. | |
| Interpretation | 20 | | Possible bias and cautious overall interpretation of results considering objectives, results from similar studies, and other relevant evidence is discussed under limitation of discussion session. | |
| Generalizability | 21 | | Generalizability (external validity) of the study results are mentioned in the paragraph of discussion section and in conclusion section. | |
| Other information | | | | |
| Funding | 22 | | Information regarding the source and extend of funding (UOG_SOM/112/7/2019) and role of funders are mentioned under declaration. | |
