# Supplementary material for: Knowledge, attitude, and practice of antenatal exercises among pregnant women in Ethiopia: A cross-sectional study
Source: PLoS One. 2021 Feb 19;16(2):e0247533. doi: 10.1371/journal.pone.0247533 (PMC7895387; doi:10.1371/journal.pone.0247533)
Supplement: S3 File — (DOCX) [file pone.0247533.s003.docx]

**Additional file 3**

# Additional tables:

Contents

[**Title: Antenatal exercises during pregnancy: knowledge, attitude, and practice of pregnant women in Ethiopia: a cross-sectional study 1**](#_Toc41935191)

[**Additional tables: 1**](#_Toc41935192)

[**Table 1 Frequency of awareness of antenatal exercises among pregnant women attending ANC at UoGCSH, Gondar, Ethiopia (n = 349) 1**](#_Toc41935193)

[**Table 2 Chi-square test of association between knowledge towards antenatal exercises and pregnant women characteristics. 2**](#_Toc41935194)

[**Table 3 Chi-square test of association between attitude towards antenatal exercises and respondents characteristics. 4**](#_Toc41935195)

[**Table 4 Chi-square test of association between practice towards antenatal exercises and respondents characteristics. 6**](#_Toc41935196)

## Table 1 Frequency of awareness of antenatal exercises among pregnant women attending ANC at UoGCSH, Gondar, Ethiopia (n = 349)

| **Characteristics** | **Frequency** | **%** |
| --- | --- | --- |
| Have you been involved in physical exercise before pregnancy  Yes  No | 127  222 | 36.4  63.6 |
| *Adviced about antenatal exercises (n = 133, 38.1%)  Relaxation therapy  Walking  Breathing exercise  Pelvic floor exercises  Ankle and toe exercises  Back care exercise  Aerobic workout  Yoga therapy  Cycling | 63  115  40  49  43  86  92  33  13 | 47.4  86.5  30.1  36.8  32.3  64.7  69.2  24.8  9.8 |
| Source of information  Health care and social workers  Family, friends, and other pregnant women  Mass media  Internet  Books | 45  31  21  24  12 | 33.8  23.3  15.8  18.0  9.0 |

*more than one item of those heard by the respondent

## Table 2 Chi-square test of association between knowledge towards antenatal exercises and pregnant women characteristics.

| Variables | IaK | AK | χ2 | P |
| --- | --- | --- | --- | --- |
| Age in years (mean 27.5 ±5.86) |  |  |  |  |
| < 25 years | 63(29.9) | 43 (31.2) | 0.45 | 0.79 |
| 25-35 years | 126 (59.7) | 78 (56.5) |  |  |
| > 35 years | 22 (10.4) | 17 (12.3) |  |  |
| Residence |  |  |  |  |
| Urban | 164 (77.7) | 126 (91.3) | 10.9 | 0.001 |
| Rural | 47 (22.3) | 12 (8.7) |  |  |
| Religion |  |  |  |  |
| Orthodox | 184 (87.2) | 113 (81.9) | 4.1 | 0.25 |
| Protestant | 04 (1.9) | 07 (5.1) |  |  |
| Muslim | 23 (10.9) | 18 (13) |  |  |
| Level of education |  |  |  |  |
| No formal educatio4n | 41 (19.4) | 10 (7.2) | 26.3 | 0.00 |
| Primary school | 30 (14.2) | 19 (13.8) |  |  |
| Secondary school | 80 (37.9) | 35 (25.4) |  |  |
| Diploma | 29 (13.7) | 36 (26.1) |  |  |
| Degree and above | 31 (14.7) | 38 (27.5) |  |  |
| Family income/month |  |  |  |  |
| < 2500 ETB | 156 (73.9) | 99 (71.7) | 6.2 | 0.04 |
| 2500-4000 ETB | 39 (18.5) | 18 (13) |  |  |
| > 4000 ETB | 16 (7.6) | 21 (15.2) |  |  |
| Type of family |  |  |  |  |
| Extended family | 18 (8.5) | 14 (10.1) | .26 | 0.6 |
| Nuclear family | 193 (91.5) | 124 (89.9) |  |  |
| Employment status |  |  |  |  |
| Unemployed | 126 (59.7) | 58 (42) | 15.2 | 0.002 |
| Governmental | 45 (21.3) | 55 (39.9) |  |  |
| Private | 15 (7.1) | 11 (8) |  |  |
| Merchant | 25 (11.8) | 14 (10.1) |  |  |
| Parity |  |  |  |  |
| No children | 86 (40.8) | 79 (57.2) | 9.12 | 0.010 |
| 1-2 children | 94 (44.5) | 45 (32.6) |  |  |
| >2 children | 31 (14.7) | 14 (10.1) |  |  |
| Gestational period |  |  |  |  |
| First trimester | 10 (4.7) | 5 (3.6) | 0.36 | 0.8 |
| Second trimester | 64 (30.3) | 40 (29) |  |  |
| Third trimester | 137 (64.9) | 93 (67.4) |  |  |
| History of miscarriage |  |  |  |  |
| Never | 186 (88.2) | 128 (92.8) | 2.7 | 0.25 |

| Once | 19 (9) | 9 (6.5) |  |  |
| --- | --- | --- | --- | --- |
| Twice and more | 6 (2.8) | 1 (0.7) |  |  |
| Previous mode of delivery |  |  |  |  |
| Labor | 101 (83.5) | 50 (76.9) | 4.6 | 0.10 |
| Caesarean | 20 (16.5) | 15 (23.1) |  |  |
| Place of delivery (n 115) |  |  |  |  |
| Hospital | 76 (63.3) | 47 (72.4) | 10.8 | 0.03 |
| Home | 31 (25.8) | 9 (13.8) |  |  |
| Hospital & home | 4 (3.3) | 0 (0) |  |  |
| PHC | 9 (7.6) | 9 (13.8) |  |  |
| Previously or currently diagnosed |  |  |  |  |
| None | 170 (80.6) | 109 (79) | 3.8 | 0.42 |
| DM | 12 (5.7) | 5 (3.6) |  |  |
| GDM | 3 (1.4) | 1 (0.7) |  |  |
| LBP | 16 (7.6) | 10 (7.2) |  |  |
| Weight gain | 10 (4.7) | 13 (9.4) |  |  |

IaK- Inadequate knowledge, AK- Adequate knowledge

## Table 3 Chi-square test of association between attitude towards antenatal exercises and respondents characteristics.

| Variables | NA | PA | χ2 | P |
| --- | --- | --- | --- | --- |
| Age in years (mean 27.5 ±5.86) |  |  |  |  |
| < 25 years | 38 (24.4) | 68 (35.2) | 10.9 | 0.004 |
| 25-35 years | 92 (59) | 112 (58) |  |  |
| > 35 years | 26 (16.7) | 13 (6.7) |  |  |
| Residence |  |  |  |  |
| Urban | 116 (74.4) | 174 (90.2) |  |  |
| Rural | 40 (25.6) | 19 (9.8) |  |  |
| Religion |  |  |  |  |
| Orthodox | 138 (88.5) | 159 (82.4) | 4.1 | 0.13 |
| Protestant | 02 (1.3) | 09 (4.7) |  |  |
| Muslim | 16 (10.3) | 25 (13) |  |  |
| Level of education |  |  |  |  |
| No formal education | 33 (21.2) | 18 (9.3) | 11.7 | 0.02 |
| Primary school | 22 (14.1) | 27 (14) |  |  |
| Secondary school | 47 (30.1) | 68 (35.2) |  |  |
| Diploma | 30 (19.2) | 35 (18.1) |  |  |
| Degree and above | 24 (15.4) | 45 (23.3) |  |  |
| Family income/month |  |  |  |  |
| < 2500 ETB | 112 (71.8) | 143 (74) | 5.3 | 0.07 |
| 2500-4000 ETB | 32 (20.5) | 25 (13) |  |  |
| > 4000 ETB | 12 (7.7) | 25 (13) |  |  |
| Type of family |  |  |  |  |

| Extended family | 14 (9) | 18 (9.3) | 0.13 | 0.91 |
| --- | --- | --- | --- | --- |
| Nuclear family | 142 (91) | 175 (90.7) |  |  |
| Employment status |  |  |  |  |
| Unemployed | 94 (60.3) | 90 (46.6) | 9.1 | 0.03 |
| Governmental | 35 (22.4) | 65 (33.7) |  |  |
| Private | 08 (5.1) | 18 (9.3) |  |  |
| Merchant | 19 (12.2) | 20 (10.4) |  |  |
| Parity |  |  |  |  |
| No children | 56 (35.9) | 109 (56.5) | 21.4 | 0.000 |
| 1-2 children | 68 (43.6) | 71 (36.8) |  |  |
| >2 children | 32 (20.5) | 13 (6.) |  |  |
| Gestational period |  |  |  |  |
| First trimester | 08 (5.1) | 07 (3.6) | 1.34 | 0.51 |
| Second trimester | 50 (32.1) | 54 (28) |  |  |
| Third trimester | 98 (62.8) | 132 (68.4) |  |  |
| History of miscarriage |  |  |  |  |
| Never | 139 (89.1) | 175 (90.7) | 2.1 | 0.35 |
| Once | 12 (7.7) | 16 (8.3) |  |  |
| Twice and more | 05 (3.2) | 02 (1) |  |  |
| Previous mode of delivery |  |  |  |  |
| Labor | 78 | 73 | 12.5 | 0.002 |
| Caesarean | 21 | 14 |  |  |
| Place of delivery (n 115) |  |  |  |  |
| Hospital | 55 (35.3) | 68 (35.2) | 22.6 | 0.000 |
| Home | 30 (19.2) | 10 (5.2) |  |  |
| Hospital & home | 02 (1.3) | 02 (1) |  |  |
| PHC | 11 (7.1) | 07 (3.6) |  |  |
| Previously or currently diagnosed |  |  |  |  |
| None | 121 | 158 | 12.3 | 0.01 |
| DM | 00 | 04 |  |  |
| GDM | 07 | 10 |  |  |
| LBP | 19 | 07 |  |  |
| Weight gain | 09 | 14 |  |  |

NA- Negative Attitude, PA- Positive Attitude

## Table 4 Chi-square test of association between practice towards antenatal exercises and respondents characteristics.

| Variables | PP | GP | χ2 | P |
| --- | --- | --- | --- | --- |
| Age in years (mean 27.5 ±5.86) |  |  |  |  |
| < 25 years | 92 (29.9) | 14 (34.1) | 1.13 | 0.56 |
| 25-35 years | 183 (59.4) | 21 (51.2) |  |  |
| > 35 years | 33 (10.7) | 06 (14.6) |  |  |
| Residence |  |  |  |  |
| Urban | 250(81.2) | 40(97.6) | 6.9 | 0.009 |
| Rural | 58(18.8) | 01(2.4) |  |  |
| Religion |  |  |  |  |
| Orthodox | 270(87.7) | 27(65.9) | 14.7 | 0.001 |
| Protestant | 07(2.3) | 04(9.8) |  |  |
| Muslim | 31(10.1) | 10(24.4) |  |  |
| Level of education |  |  |  |  |
| No formal education | 49(15.9) | 02(4.9) | 20.7 | 0.000 |
| Primary school | 47(15.3) | 02(4.9) |  |  |
| Secondary school | 105(34.1) | 10(24.4) |  |  |
| Diploma | 56(18.2) | 09(22) |  |  |
| Degree and above | 51(16.6) | 18(43.9) |  |  |
| Family income/month |  |  |  |  |
| < 2500 ETB | 241(78.1) | 13(31.) | 72.3 | 0.000 |
| 2500-4000 ETB | 49(15.9) | 08(19.5) |  |  |
| > 4000 ETB | 18(5.8) | 20(48.8) |  |  |
| Type of family |  |  |  |  |
| Extended family | 29(9.4) | 03(7.3) | 0.19 | 1.11 |
| Nuclear family | 279(90.6) | 38(92.7) |  |  |
| Employment status |  |  |  |  |
| Unemployed | 170(55.2) | 14(34.1) | 11.7 | 0.008 |
| Governmental | 85(27.6) | 15(36.6) |  |  |
| Private | 24(7.8) | 02(4.9) |  |  |
| Merchant | 29(9.4) | 10(24.4) |  |  |
| Parity |  |  |  |  |
| No children | 134(43.5) | 31(75.6) | 16.6 | 0.000 |
| 1-2 children | 129(41.9) | 10(24.4) |  |  |
| >2 children | 45(14.6) | 0(0) |  |  |
| Gestational period |  |  |  |  |
| First trimester | 13(4.2) | 02(4.9) | 0.21 | 0.89 |
| Second trimester | 93(30.2) | 11(26.8) |  |  |
| Third trimester | 202(65.6) | 28(68.3) |  |  |
| History of miscarriage |  |  |  |  |
| Never | 278(90.3) | 36(87.8) | 1.96 | 0.37 |
| Once | 23(7.5) | 05(12.2) |  |  |
| Twice and more | 072.3() | 0(0) |  |  |
| Previous mode of delivery |  |  |  |  |
| Labor | 137 | 14 | 2.6 | 0.26 |
| Caesarean | 32 | 03 |  |  |
| Place of delivery (n 115) |  |  |  |  |
| Hospital | 109 | 14 | 4.9 | 0.29 |
| Home | 37 | 03 |  |  |
| Hospital & home | 140 | 0 |  |  |
| Previously or currently diagnosed |  |  |  |  |
| None | 252(81.8) | 27(65.9) | 16.1 | 0.003 |
| DM | 13(4.2) | 04(9.8) |  |  |
| GDM | 04(1.3) | 0(0) |  |  |
| LBP | 24(7.8) | 02(4.9) |  |  |
| Weight gain | 15(4.9) | 08(19.5) |  |  |
| PHC | 18 | 0 |  |  |

NA- Good Practice, PP- Poor Practice
