# Supplementary material for: Knowledge, attitude, and practice of antenatal exercises among pregnant women in Ethiopia: A cross-sectional study
Source: PLoS One. 2021 Feb 19;16(2):e0247533. doi: 10.1371/journal.pone.0247533 (PMC7895387; doi:10.1371/journal.pone.0247533)
Supplement: Attachment — Submitted filename: Response to reviewers.docx [file pone.0247533.s004.docx]

**Date: January 2021**

# POINT-BY-POINT RESPONSE LETTER

PLOS ONE

# Manuscript ID PONE-D-20-30016 Title: Knowledge, attitude, and practice of antenatal exercises among pregnant women in Ethiopia: a cross-sectional study We express our sincere appreciation to the reviewers and editor for their time, comments, suggestions, and interest in this study. The reviewers and editor had pointed out constructive comments, and legitimate questions which improved the paper.

Please find our responses below:

***Note: Correction and edition made in the track change version:***

|  | **(Reviewer-1) Review questions/comments** | **Response** |
| --- | --- | --- |
| **1** | The authors evaluated the knowledge, attitudes, and practice of antenatal exercise and association with the socio-demographic and maternal characteristics among Ethiopian pregnant women. This is an important, but neglected subject in maternal health, particularly in African context. The findings of the study are worthwhile to inform policy direction and action in crafting intervention to address the gaps pertaining to the knowledge, attitudes and practice of prenatal physical activity. I have few minor comments as follows | **Thank you, for your comments and time consideration.**  **We have amended the corrections recommended and pointed.** |
| **2.** | Abstract 1. The objective of this study was to evaluate the knowledge, attitude, and practice of antenatal exercises among Ethiopian women during pregnancy, and also to find out why some women do not exercise during pregnancy” I suggest change the phrase ‘to find out why”… “to examine the barriers to prenatal physical activity”. | Thank you changes amended accordingly  Page 02, Abstract background section, line no 25-26 |
| **3.** | “ANC” Write in full | Done accordingly  Page 02, Abstract section, method sub-section, 1^st^ para line no 28 |
| **4.** | ”among them had a good practice” Revise this phrase. I suggest changing among them to pregnant women. | **Thank you, revised accordingly**  Page 02, Abstract section, results sub-section, line no 33 |
| **5.** | “More than half of them (53.6) think that ANEx doesn’t suit Ethiopian culture” . Again see my earlier comment in 3 above concerning them. Furthermore, write formally. Change ‘doesn’t’. The word ‘suit’ is not talking well to the context. | **Yes, accepted and revised accordingly**  Page 02, Abstract section, results sub-section, line no 36-37. |
| **6.** | Methodology “Pregnant mothers aged between 16 and 49 years, during any trimester of pregnancy those who were…” There is something missing in the sentence. | The sentence is rephrased now  Page no 7, Method section, 3^rd^ Para, line no 126-128 |
| **7.** | Results  “The education tally showed..” Rephrase this phrase | The sentence is rephrased now  Page no 10, result section, line no 201-202 |
| **8.** | “Walking is the most accomplished” The word accomplished is not appropriate in the context. | Thank you, changed accordingly  Page no 14, result section, line no 246-247 |
| **9.** | “The foremost barriers to do ANEx reported by women not engaged in exercise were being afraid that ANEx could be…” This sentence need editing. | The sentence is copy edited now  Page no 15, result section, line no 248-249 |
| **10.** | “In multivariate analyses when adjusted for the other independent variables, better education was a significant predictor of the knowledge of ANEx among pregnant mother”. What is better education? | We meant higher education and the changes are amended now accordingly, thank you for pointing out.  Page no 15, Result section- Determinants of KAP subsection, 1^st^ para, line no 256 |
| **11.** | Duplicate references removed | Reference no: 36, 33, 38, 42, 39 |

With regards

Balamurugan Janakiraman

[bala77physio@gmail.com](mailto:bala77physio@gmail.com)
